# 40339 Characterizing the impact of social and structural determinants of health on racial and ethnic disparities in COVID-19 outcomes using electronic health record (EHR) data

**DOI:** 10.1017/cts.2021.475

**Published:** 2021-03-30

**Authors:** Lauren Heery, Kristine Erlandson, Krithika Suresh, Karen Albright, Lisa Schilling

**Affiliations:** University of Colorado Anschutz Medical Campus

## Abstract

ABSTRACT IMPACT: This study will help to characterize the root causes of racial and ethnic disparities in viral respiratory outbreaks and determine the extent to which this is unique to the COVID-19 pandemic, so that preventative interventions can be designed for future pandemics and epidemics. OBJECTIVES/GOALS: The causes of racial and ethnic disparities in COVID-19 clinical outcomes are multifactorial but include social inequity driven by structural racism. This study seeks to characterize the patterns of these disparities by linking patient-level EHR data with population-level sociodemographic measures. METHODS/STUDY POPULATION: This retrospective review of adult patients tested for SARS-CoV-2 in the UCHealth System will compare rates of COVID-19 infection, hospitalization, in-hospital mortality and 30-day mortality across racial and ethnic groups. Race and ethnicity are determined by patient self-report in the EHR. Univariable and multivariable regression analyses will be used to assess the association of these outcomes with sociodemographic factors. Potential confounders that will be adjusted for include Charlson Co-morbidity Index, disease severity and likelihood of readmission. Using chi-square tests, we will assess differences in the race/ethnicity distributions between this cohort and those from the 2009 H1N1 Pandemic and the 2018-19 influenza season. RESULTS/ANTICIPATED RESULTS: Of the first 459 patients hospitalized for COVID-19 in March and April 2020, race/ethnicity were: 194 Hispanic (42.3%), 104 non-Hispanic Black (22.6%), 83 non-Hispanic white (18.1%), 43 Asian (9.4%), and 35 other or unknown race (7.6%). There were significant differences in the race/ethnicity distribution compared to the cohort of patients hospitalized for viral respiratory infection during the 2018-19 influenza season (n=254, p <0.001): 58 Hispanic (22.8%), 52 non-Hispanic black (20.4%), 116 non-Hispanic white (45.7%), 15 Asian (6%), and 13 other or unknown race (5.1%). Our anticipated results include further adjusted analyses and comparisons to the 2009 pandemic. We will compare COVID-19 prevalence and outcomes by race/ethnicity with other viral infection outbreaks, adjusting for confounders. DISCUSSION/SIGNIFICANCE OF FINDINGS: Initial hospitalizations for COVID-19 at our institution are notable for a high proportion of Hispanic patients and smaller proportion of non-Hispanic whites, in contrast to the prior year. Our study will demonstrate the extent to which racial and ethnic disparities are typical in viral respiratory outbreaks, which can guide future interventions.

